# Flexible polarimetric probe for 3 × 3 Mueller matrix measurements of biological tissue

**DOI:** 10.1038/s41598-017-12099-8

**Published:** 2017-09-20

**Authors:** Sarah Forward, Adam Gribble, Sanaz Alali, Andras A. Lindenmaier, I. Alex Vitkin

**Affiliations:** 1 University of Toronto, Department of Medical Biophysics, Toronto, ON M5G 1L7 Canada; 2 ASML Wilton, Wilton, CT 06897 USA; 3 University Health Network, Toronto, ON M5G 1L7 Canada

## Abstract

Polarimetry is a noninvasive method that uses polarised light to assess biophysical characteristics of tissues. A series of incident polarisation states illuminates a biological sample, and analysis of sample-altered polarisation states enables polarimetric tissue assessment. The resultant information can, for example, help quantitatively differentiate healthy from pathologic tissue. However, most bio-polarimetric assessments are performed using free-space optics with bulky optical components. Extension to flexible fibre-based systems is clinically desirable, but is challenging due to polarisation-altering properties of optical fibres. Here, we propose a flexible fibre-based polarimetric solution, and describe its design, fabrication, calibration, and initial feasibility demonstration in *ex vivo* tissue. The design is based on a flexible fibre bundle of six multimode optical fibres, each terminated with a distal polariser that ensures pre-determined output polarisation states. The resultant probe enables linear 3 × 3 Mueller matrix characterization of distal tissue. Potential *in vivo* Mueller matrix polarimetric tissue examinations in various directly-inaccessible body cavities are envisioned.

## Introduction

The use of polarised light to assess and quantify tissue biophysical properties noninvasively and without exogenous contrast agents is known as tissue polarimetry. Biological components (e.g., cells and their nuclei, collagen fibres, blood capillaries) alter the polarisation of incident light upon interaction, as specified by the geometry and inherent optical properties of these heterogeneous scattering structures^1^. The most complete polarimetry description is the sample’s Mueller matrix, a 16-element (4 × 4) polarimetric transfer function of the probed tissue. Useful biophysical quantities derived from this Mueller matrix, such as linear retardance (a measure of tissue anisotropy), can reveal precursors of malignant disease related to connective tissue and fibrillar protein morphology^2^, while depolarisation contrast (a measure of tissue micro-organization) can help identify tumour margins in pre-clinical breast cancer models^3^.

Bulky polarisation optics and lenses limit traditional free-space polarimetry to studies of *ex vivo* tissue, histology slides, or easily and directly accessible sites such as human skin. However, *in vivo* examination of various mucosal tissue linings where much pathology originates is paramount for early detection and surveillance in oncology and beyond (e.g., colon cancer^4^, bladder cancer^5^, and Barrett’s oesophagus^6^). These important clinical sites are currently inaccessible to Mueller matrix examinations, because the flexible endoscopes necessary to reach and probe these sites contain optical fibres that greatly distort the polarisation information upon bending. Thus, the often small and subtle tissue polarimetric signature would be overwhelmed by system-delivery polarisation artefacts. Polarisation-maintaining fibres offer a possible way to guide polarised light, but these are specific to one polarisation and suffer from low single mode fibre numerical aperture, limiting the light collected back from tissue.

Several research groups are actively pursuing novel solutions to reduce such system artefacts and enable true distal tissue polarimetry. For example, Myakov *et al*. and Turzhitsky *et al*. have constructed flexible fibre-based probes, however they are only used for polarised reflectance spectroscopy^7,8^, and are unable to extract the Mueller matrix of tissue necessary to yield its full polarisation signature. Vizet *et al*. have reported a flexible fibre-based probe capable of measuring basic polarisation properties^9^, however the light collection efficiency is low as limited by single mode fibre acceptance angle (numerical aperture NA = 0.12), requiring sensitive detectors; further, the multi-wavelength use introduces the issue of different wavelength photons interacting within different tissue sampling volumes/depths. Their recent advance^10^ uses a dual-wavelength approach where the polarisation effect of the fibre must be characterized at one wavelength and decoupled from the sample Mueller matrix measured at the other wavelength. Qi and Elson have made a polarisation-resolved endoscope capable of full wide-field 4 × 4 Mueller imaging^11^, but their approach is limited to rigid, forward-viewing endoscopy. The rigidity provides certain inherent advantages such as technological simplicity, longer shelf life, low chromatic aberrations, and ease of manoeuvring. However their utility is limited to relatively accessible clinical sites, so they are often not feasible for investigation of difficult-to-reach inner cavities such as the bladder, colon, airways, and upper gastrointestinal tract.

As *flexible* endoscopy is the clinical standard for examinations of internal body surface linings and cavities, the inherent and varying birefringence of flexible single- and multimode optical fibres must be overcome. This birefringence causes alterations in the polarisation of transmitted light that are often unpredictable and amplified by fibre bending. Here we propose an alternative, single-wavelength photonic design compatible with flexible probes using high light collection efficiency multimode optical fibres (fibre NA = 0.39) terminated with distal polarising elements. The distal location of the polarisers ensures that the light incident on the tissue has a user-defined, known polarisation state, and the polarisation analyser characteristics that detect tissue-reflected light are similarly known. The inevitable fibre artefact distortions still exist, but they now manifest as variations in incident light intensity instead of variations in polarisation, and can be accounted for with careful calibration, as described in section 2.3.

The described six-fibre probe is small enough to fit through and bend with the working channels of commercial flexible cystoscopes and colonoscopes (diameter ~3mm), to offer a potential polarimetric enhancement to such endoscopies. At present the probe implementation is limited to measuring a 9-element (3 × 3) Mueller matrix, as it uses linear polarisers only. It is possible to incorporate quarter wave plates (QWP) for circular polarisation measurements, and hence measure the full 4 × 4 16-element Mueller matrix. Here for proof of concept, linear polarisers were used exclusively.

In this paper we report on our linear Mueller probe design, fabrication, approach for data acquisition and calibration, and performance in probing known optical polarisation samples and *ex vivo* birefringent biological tissues.

## Materials and Methods

### Probe Design and Fabrication

The probe uses six multimode, 400-μm-diameter core fibres for light delivery or collection (Thorlabs M28L01, NA = 0.39, refractive index (RI) = 1.457 at 635 nm, cut in half and cleaved straight, leaving an SMA adapter on one end). A ~30 cm fibre length was employed for this particular probe embodiment; different fibre lengths can be used, e.g. several metres long to accommodate gastro-intestinal endoscopy, with similar negligible losses. To determine a 9-element Mueller matrix uniquely, at least 9 different measurements must be performed using all input/output combinations of 3 different linear polarisations^12^. Since we do not illuminate and collect light through the same fibre simultaneously (for this proof-of-principle prototype only; the use of circulators will enable this in future probe versions^13,14^) 2 fibres are needed per polarisation, hence 6 fibres were used.

**Figure 1:**
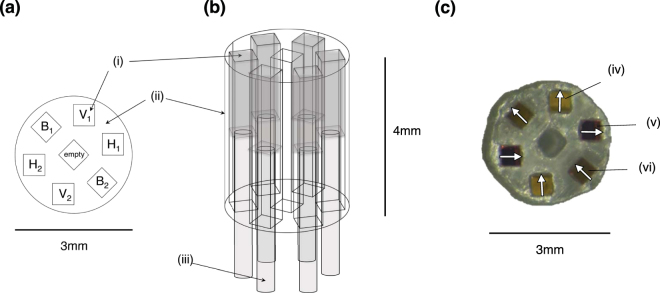
(**a**) Top view of 3-dimensional schematic of the printed component holder with six 500 μm × 500 μm micro-polariser pieces (i), oriented vertically (labelled V_1_ and V_2_), horizontally (H_1_ and H_2_), and at −45° (B_1_ and B_2_) in the holder (ii). (**b**) Side view of 3-dimensional printed component holder design, with optical fibres (iii) glued to the underside of each micro-polariser (i). (**c**) Top view of experimental 3D printed component holder with V, H and B micro-polarisers placed into position; the arrows represent their orientations. The photograph was taken through a vertical polariser sheet with a stereomicroscope; thus light reflected back off the white stereomicroscope stage through the horizontal polariser (v) is blocked, the light through the −45° polariser suffers a ~50% intensity loss (vi), and the vertical polariser intensity (iv) is largely unaffected. Other fibre geometries are possible to house the channels closer together, improving resolution, including arrangements that make use of the central channel. The depicted geometry was suitable for a proof-of-principle prototype demonstration.

The micron-scale polarisers (‘micro-polarisers’) were manufactured by cutting a linear polariser plate (Thorlabs LPVISB050, 2 mm thickness, RI = 1.52 at 635 nm, 10,000:1 extinction ratio) with an automatic dicing saw (Disco DAD3220, 100-μm-thick diamond blade) into 500 μm × 500 μm squares (to adequately cover the whole fibre cross-section). The polariser was cut into squares with edges aligned with its transmission axis for easy orientation; circular cuts would have made it more difficult to keep track of the transmission axes, and more difficult to assemble with the desired orientations. A holder with square channels was 3D-printed (MED610 photopolymer material, Stratasys PolyJet printer) to house and maintain relative alignment of the micro-polarisers, which were placed in the 3D-printed holder, and then the cleaved ends of the optical fibres were attached. The fibres were glued to each micro-polariser using Norland #85 glue (RI = 1.46 in visible range) and cured with a UVB light source (Dymax). This glue had the closest index of refraction to both the fibre and polariser so as to minimize reflections, but it wasn’t strong enough on its own to reliably hold both components together. Thus, a stronger glue (Dymax 203-CTH-F-T, RI = 1.58 in visible range) was applied overtop, to reinforce the bond, and was cured with the same lamp. The probe’s schematic arrangement and resultant experimental prototype are shown in Fig. 1.

**Figure 2:**
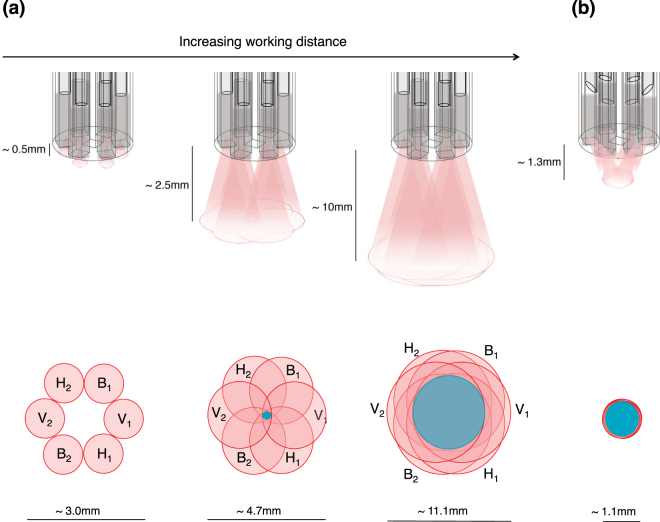
(**a**) The relationship between probe-tissue working distance and surface illumination/detection profiles. These profiles are estimated using the fibre NA; actual illumination profiles will be slightly wider as light also passes through the 2 mm thick polariser elements and slightly scatters through the component holder. The blue coloured regions indicate the approximate surface profile overlap from the 6 fibres. In reality due to volumetric tissue scattering, light spreads beyond the irradiance spots and exhibits greater commonality of the sampling volumes; these surface contours merely represent a first-order approximation. Various methods of photon profile sculpting, such as fibre tip bevelling (**b**) to steer the illumination spots, and ball lenses ref.^33^ for focusing, will be used in pre-clinical/clinical prototypes to optimize the working distance, sampling volume, and photon budget parameter space.

The MED610 photopolymer allowed for hollow channels to be printed within nearly 500 μm of each other; any closer and the material would crumble. To illuminate and collect light from overlapping tissue volumes, the probe’s geometry was thus designed with this fibre-to-fibre separation in mind. Of course, the sampling volume/field of view (FOV) and degree of sampling overlap between different probe fibres also depends on the working distance between the probe and tissue, as illustrated in Fig. 2. The illumination/detection spots shown in the bottom row of the figure only depict surface effects; in biological tissues volumetric multiple scattering spreads the photon flux considerably beyond the surface profiles, so in reality the overlaps are somewhat greater. In general, decreasing the working distance lowers the FOV/sampling volume, and also increases the possibility of different probe fibres interrogating slightly different (partially overlapping) tissue regions. Conversely, larger working distances will ensure better overlap between fibres and increase the effective tissue sampling volume, but will decrease resolution and likely cause photon budget problems. Detailed polarisation-sensitive Monte Carlo simulations of light transport in tissue^15^ will examine this interconnected parameter space for future probe implementations.

### Mathematical Description

The three orientations of the micro-polarisers used in the probe sufficiently span the linear polarisations (this is analogous to spanning the Poincaré sphere^16,17^ in 4 × 4 Mueller polarimetry), allowing robust determination of the 3 × 3 linear Mueller matrix via 9 intensity-based measurements. These 9 measurements can be modelled by a Stokes vector formalism that describes the light polarisation by

1 $${\boldsymbol{S}}=\,[\begin{array}{c}I\\ Q\\ U\\ V\end{array}]=[\begin{array}{c}{I}_{0}+{I}_{90}\\ {I}_{0}-{I}_{90}\\ {I}_{45}-{I}_{-45}\\ {I}_{R}-{I}_{L}\end{array}],$$

where *I* is the overall intensity of the light (sum of any two orthogonal polarisation states), *Q* is the difference between the intensity of horizontally (0°) and vertically (90°) polarised light, *U* is the difference between the intensity of +45° and −45° polarised light, and *V* is the difference between the intensity of right (R) and left (L) circularly polarised light; note that we don’t pursue the *V* parameter in our current experimental demonstration of the linear Mueller matrix probe. Vectors and matrices are represented in bold. The tissue Mueller matrix ***M*** is calculated using

2 $${{\boldsymbol{S}}}_{{\boldsymbol{OUTPUT}}}={\boldsymbol{M}}\cdot {{\boldsymbol{S}}}_{{\boldsymbol{INPUT}}},$$

where ***S***_***INPUT***_ and ***S***_***OUTPUT***_ are the input (pre-sample interaction) and output (post sample interaction) Stokes vectors. The Mueller matrix is the transfer function of the sample, representing its complete polarisation fingerprint. Once determined, intrinsic tissue biophysical polarisation properties such as retardance, diattenuation, and depolarisation can be obtained from it via polar decomposition^18,19^.

Here we use the following 9 probe measurements to determine the Mueller matrix of a sample: H_1_H_2_, H_1_V_2_, H_1_B_1_, V_2_H_1_, V_2_V_1_, V_2_B_1_, B_1_H_1_, B_1_V_2_, and B_1_B_2_, where the first letter and number indicates which channel is used to illuminate, and the second indicates which channel is used to collect. Note we do not perform the measurements in redundancy here to improve SNR (e.g. determine the HV data from H_1_V_2_, H_1_V_1_, H_2_V_2_, and H_2_V_1_ measurements) for mathematical simplicity in our proof-of-principle probe.

Following Eq. (2), for a sample that is interrogated by an arbitrary pair of excitation and collection channels, the resultant detected polarisation vector, ***S***_***OUTPUT***_, is:

3 $${{\boldsymbol{S}}}_{{\boldsymbol{OUTPUT}}}={{\boldsymbol{M}}}_{{COLLECTION}{FIBRE}}\cdot {{\boldsymbol{M}}}_{{\boldsymbol{MICRO}}-{\boldsymbol{POLARISER}}}\cdot {{\boldsymbol{M}}}_{{\boldsymbol{SAMPLE}}}\cdot {{\boldsymbol{S}}}_{{\boldsymbol{INPUT}}},$$

where ***S***_***INPUT***_ is the Stokes vector incident on the sample, and ***S***_***OUTPUT***_ is the polarisation state exiting the proximal end of the collection fibre (whose intensity will be measured with a photodetector in our data acquisition scheme). As per Eq. (3), ***S***_***INPUT***_ is transformed into ***S***_***OUTPUT***_ by the product of the sample’s Mueller matrix, the Mueller matrix of the collection micro-polariser, and the Mueller matrix of the collection fibre.

The 9 intensity measurements of the sample are equivalent to the *I*s (total intensity) Stokes parameters of each respective ***S***_***OUTPUT***_; that is, we need only know the *I* Stokes parameter of the collected light in determining tissue sample’s Mueller matrix. Note that since the current probe’s fibres are ~30 cm long, we assume the total intensity of collected light is conserved after traversing the distal micro-polariser, with negligible losses through the collection fibre. Therefore, we neglect the inconsequential effect of ***M***_***COLLECTION FIBRE***_ on the total intensity of ***S***_***OUTPUT***_. Thus, Eq. (3) reduces to:

4 $${I}_{{\boldsymbol{OUTPUT}}}=\frac{1}{2}[\begin{array}{ccc}{p}_{11} & {p}_{12} & {p}_{13}\end{array}]\cdot {{\boldsymbol{M}}}_{{\boldsymbol{SAMPLE}}}\cdot {{\boldsymbol{S}}}_{{\boldsymbol{INPUT}}},$$

where *I*_***OUTPUT***_ is the total intensity of ***S***_***OUTPUT***_ at the proximal end of the collection fibre and ½[p_11_ p_12_ p_13_] is the first row of the Mueller matrix of the collection fibre’s micro-polariser. The second and third row of ***M***_***MICRO-POLARISER***_ do not contribute to the magnitude of *I*_***OUTPUT****.*_

For an ideal linear polariser, the first row elements of its Mueller matrix, [p_11_ p_12_ p_13_] are equivalent to the normalized Stokes parameters, [*I Q U*], of light whose polarisation is parallel to the ideal polariser’s transmission axis. Since we can’t measure ***M***_***MICRO-POLARISER***_ directly (i.e., the Stokes vectors of light that pass through the micro-polariser into the collection fibre are not accessible since they are altered by the polarisation properties of the fibre, before reaching a detector), we use the Stokes parameters of light emerging *out* the micro-polariser into free space and reverse the sign of the *U* parameter (the Stokes vector of light emanating *out* of, *vs. after* the micro-polariser, differ by 180° −θ in the reference frame of travelling light, thus corresponding to a sign change of *U*). Therefore we represent the first row of the micro-polariser’s Mueller matrix by:

5 $$\frac{1}{2}[\begin{array}{ccc}{p}_{11} & {p}_{12} & {p}_{13}\end{array}]=\frac{1}{2}[\begin{array}{ccc}I & Q & -U\end{array}],$$

where *U* is what emerges from the micro-polariser, and –*U* is what would be collected.

For example, the *H*_1_ *H*_2_ intensity measurement can be expressed as:

6 $${H}_{1}{H}_{2}=\frac{1}{2}[\begin{array}{ccc}{I}_{H2} & {Q}_{H2} & -{U}_{H2}\end{array}].[\begin{array}{ccc}{m}_{11} & {m}_{12} & {m}_{13}\\ {m}_{21} & {m}_{22} & {m}_{23}\\ {m}_{31} & {m}_{32} & {m}_{33}\end{array}].[\begin{array}{c}{I}_{H1}\\ {Q}_{H1}\\ {U}_{H1}\end{array}],$$

where *m*_*ij*_ are the i^th^-row and j^th^-column elements of ***M***_***SAMPLE***_ and the subscripts on *I*, *Q*, and *U* denote which probe channel they are associated with. Multiplying and rearranging:

7 $${H}_{1}{H}_{2}=\frac{1}{2}(\begin{array}{ccc}{I}_{H2}.[{I}_{H1}\,{Q}_{H1}\,{U}_{H1}] & \,{Q}_{H2}.[{I}_{H1}\,{Q}_{H1}\,{U}_{H1}] & -\,{U}_{H2}.[{I}_{H1}\,{Q}_{H1}\,{U}_{H1}]\end{array}).[\begin{array}{c}{m}_{11}\\ {m}_{12}\\ {m}_{13}\\ {m}_{21}\\ {m}_{22}\\ {m}_{23}\\ {m}_{31}\\ {m}_{32}\\ {m}_{33}\end{array}]$$

The H_1_H_2_ measurement makes up 1 out of 9 intensity measurements required to determine the linear 3 × 3 Mueller matrix of a sample. A similar equation can also be written for each of the 8 remaining intensity measurements (H_1_V_2_, H_1_B_1_, V_2_H_1_, etc.). Together, these 9 equations allow a unique solution for the Mueller matrix. Due to the slow manual switching of fibres with the current probe, these 9 measurements take approximately 5 minutes to perform. Grouping these equations together, the following expression is derived:

8 $$[\begin{array}{c}{H}_{1}{H}_{2}\\ \begin{array}{c}{H}_{1}{V}_{2}\\ {H}_{1}{B}_{1}\end{array}\\ \begin{array}{c}{V}_{2}{H}_{1}\\ {V}_{2}{V}_{1}\end{array}\\ \begin{array}{c}{V}_{2}{B}_{1}\\ \begin{array}{c}{B}_{1}{H}_{1}\\ {B}_{1}{V}_{2}\end{array}\end{array}\\ {B}_{1}{B}_{2}\end{array}]=\frac{1}{2}[\begin{array}{ccc}{I}_{H2}{I}_{H1} & \ldots  & -{U}_{H2}{U}_{H1}\\ \vdots  & \ddots  & \vdots \\ {I}_{B2}{I}_{B1} & \ldots  & -{U}_{B2}{U}_{B1}\end{array}].[\begin{array}{c}{m}_{11}\\ {m}_{12}\\ {m}_{13}\\ {m}_{21}\\ {m}_{22}\\ {m}_{23}\\ {m}_{31}\\ {m}_{32}\\ {m}_{33}\end{array}]$$

where the abbreviated matrix above (we refer to this matrix of measured Stokes parameters as the *system matrix* ‘***Z***’) in its full form is:

9 $${\boldsymbol{Z}}=[\begin{array}{ccccccccc}{I}_{H2}{I}_{H1} & {I}_{H2}{Q}_{H1} & {I}_{H2}{U}_{H1} & {Q}_{H2}{I}_{H1} & {Q}_{H2}{Q}_{H1} & {Q}_{H2}{U}_{H1} & -{U}_{H2}{I}_{H1} & -{U}_{H2}{Q}_{H1} & -{U}_{H2}{U}_{H1}\\ {I}_{V2}{I}_{H1} & {I}_{V2}{Q}_{H1} & {I}_{V2}{U}_{H1} & {Q}_{V2}{I}_{H1} & {Q}_{V2}{Q}_{H1} & {Q}_{V2}{U}_{H1} & -{U}_{V2}{I}_{H1} & -{U}_{V2}{Q}_{H1} & -{U}_{V2}{U}_{H1}\\ {I}_{B1}{I}_{H1} & {I}_{B1}{Q}_{H1} & {I}_{B1}{U}_{H1} & {Q}_{B1}{I}_{H1} & {Q}_{B1}{Q}_{H1} & {Q}_{B1}{U}_{H1} & -{U}_{B1}{I}_{H1} & -{U}_{B1}{Q}_{H1} & -{U}_{B1}{U}_{H1}\\ {I}_{H1}{I}_{V2} & {I}_{H1}{Q}_{V2} & {I}_{H1}{U}_{V2} & {Q}_{H1}{I}_{V2} & {Q}_{H1}{Q}_{V2} & {Q}_{H1}{U}_{V2} & -{U}_{H1}{I}_{V2} & -{U}_{H1}{Q}_{V2} & -{U}_{H1}{U}_{V2}\\ {I}_{V1}{I}_{V2} & {I}_{V1}{Q}_{V2} & {I}_{V1}{U}_{V2} & {Q}_{V1}{I}_{V2} & {Q}_{V1}{Q}_{V2} & {Q}_{V1}{U}_{V2} & -{U}_{V1}{I}_{V2} & -{U}_{V1}{Q}_{V2} & -{U}_{V1}{U}_{V2}\\ {I}_{B1}{I}_{V2} & {I}_{B1}{Q}_{V2} & {I}_{B1}{U}_{V2} & {Q}_{B1}{I}_{V2} & {Q}_{B1}{Q}_{V2} & {Q}_{B1}{U}_{V2} & -{U}_{B1}{I}_{V2} & -{U}_{B1}{Q}_{V2} & -{U}_{B1}{U}_{V2}\\ {I}_{H1}{I}_{B1} & {I}_{H1}{Q}_{B1} & {I}_{H1}{U}_{B1} & {Q}_{H1}{I}_{B1} & {Q}_{H1}{Q}_{B1} & {Q}_{H1}{U}_{B1} & -{U}_{H1}{I}_{B1} & -{U}_{H1}{Q}_{B1} & -{U}_{H1}{U}_{B1}\\ {I}_{V2}{I}_{B1} & {I}_{V2}{Q}_{B1} & {I}_{V2}{U}_{B1} & {Q}_{V2}{I}_{B1} & {Q}_{V2}{Q}_{B1} & {Q}_{V2}{U}_{B1} & -{U}_{V2}{I}_{B1} & -{U}_{V2}{Q}_{B1} & -{U}_{V2}{U}_{B1}\\ {I}_{B2}{I}_{B1} & {I}_{B2}{Q}_{B1} & {I}_{B2}{U}_{B1} & {Q}_{B2}{I}_{B1} & {Q}_{B2}{Q}_{B1} & {Q}_{B2}{U}_{B1} & -{U}_{B2}{I}_{B1} & -{U}_{B2}{Q}_{B1} & -{U}_{B2}{U}_{B1}\end{array}].$$

This system matrix can be determined by measuring the normalized Stokes vectors of light emanating from each channel. This probe characterization step needs only to be done once (after manufacturing of the probe, before the calibration described later); the normalized Stokes vectors of light exiting each channel will *not* change with fibre bending. For improved accuracy, we do not assume cardinal angle orientations (−45, 0, and 90 degrees) of the micro-polarisers, but rather measure these independently (Thorlabs PAX5710VIS-T free-space polarimeter, ±0.25° orientation accuracy).

Thus, by measuring these Stokes parameters to construct the system matrix, and using the probe to measure the 9 intensities after interaction with an unknown (tissue) sample, all nine elements of the sample’s 3 × 3 Mueller matrix can be determined through inversion of Eq. (8):

10 $$[\begin{array}{c}{m}_{11}\\ {m}_{12}\\ {m}_{13}\\ {m}_{21}\\ {m}_{22}\\ {m}_{23}\\ {m}_{31}\\ {m}_{32}\\ {m}_{33}\end{array}]=2\cdot {{\boldsymbol{Z}}}^{-1}\cdot [\begin{array}{c}{H}_{1}{H}_{2}\\ \begin{array}{c}{H}_{1}{V}_{2}\\ {H}_{1}{B}_{1}\end{array}\\ \begin{array}{c}{V}_{2}{H}_{1}\\ {V}_{2}{V}_{1}\end{array}\\ \begin{array}{c}{V}_{2}{B}_{1}\\ \begin{array}{c}{B}_{1}{H}_{1}\\ {B}_{1}{V}_{2}\end{array}\end{array}\\ {B}_{1}{B}_{2}\end{array}].$$

Eq. (10) is valid if the 6 probe channels all couple light equally (both inter- and intra-channel). Since this is likely not the case in practice, we must calibrate the probe. Our calibration procedure (see below) generates 9 coefficients (c_1_, c_2_, …, c_9_) that scale each of the 9 experimental sample intensity measurements to account for coupling differences. This turns Eq. (10) into:

11 $$[\begin{array}{c}{m}_{11}\\ {m}_{12}\\ {m}_{13}\\ {m}_{21}\\ {m}_{22}\\ {m}_{23}\\ {m}_{31}\\ {m}_{32}\\ {m}_{33}\end{array}]=2\cdot {{\boldsymbol{Z}}}^{-1}\cdot [\begin{array}{c}{c}_{1}\cdot {H}_{1}{H}_{2}\\ {c}_{2}\cdot {H}_{1}{V}_{2}\\ \begin{array}{c}{c}_{3}\cdot {H}_{1}{B}_{1}\\ {c}_{4}\cdot {V}_{2}{H}_{1}\end{array}\\ \begin{array}{c}{c}_{5}\cdot {V}_{2}{V}_{1}\\ \begin{array}{c}{c}_{6}\cdot {V}_{2}{B}_{1}\\ {c}_{7}\cdot {B}_{1}{H}_{1}\end{array}\end{array}\\ {c}_{8}\cdot {B}_{1}{V}_{2}.\\ {c}_{9}\cdot {B}_{1}{B}_{2}\end{array}].$$

The coefficients c_i_ take into account the factors of fibre coupling efficiency, inter-channel geometry (see Fig. 2), and possible slight internal reflections at glued interfaces induced by refractive index mismatches. For instance, consider the case of the probe set up directly facing a mirror. For this case H_1_H_2_ and V_1_V_2_ should give identical results. If the intensity of the H_1_H_2_ measurement was measured to be ¾ of that of the V_1_V_2_ measurement, then the calibration coefficient for H_1_H_2_ would be ~ 4/3 that of the coefficient for V_1_V_2_. Further, the intensity of light that emerges from each fibre will vary depending on its micro-polariser orientation and if/how the fibre is bent. For example, despite our efforts to depolarise the laser source before coupling into the probe fibres, the light was still partially polarised (degree of polarisation (DOP) = ~15%); thus the orientation of this small DOP with respect to the micro-polarisers will influence the intensity of light emerging from each channel. The calibration procedure to determine the c_i_ coefficients is thus necessary, as these various sample-independent experimental factors can affect the detected light intensity and thus distort the determined tissue Mueller matrix results.

### Calibration Procedure

**Figure 3:**
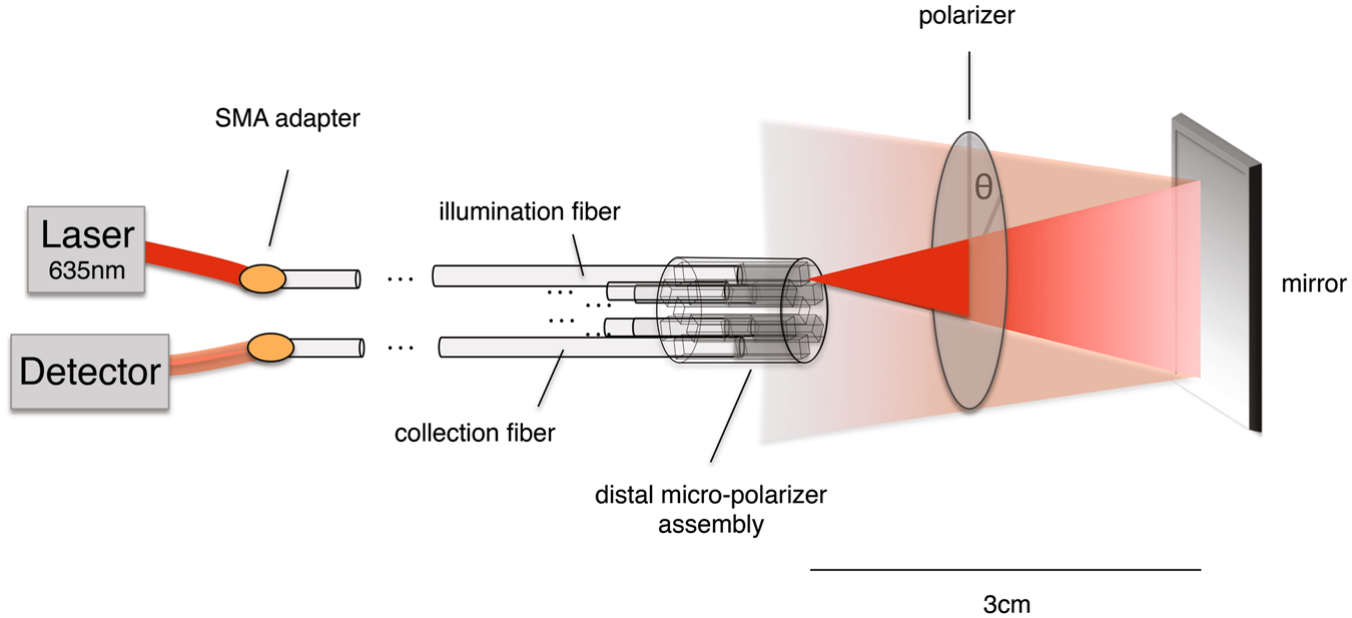
Probe calibration setup. The probe’s distal end faces an external linear polariser followed by a front-surface mirror. All 9 combinations of illumination/detection channels were sampled by appropriate switching at the probe’s proximal end. Three polariser orientation angles of θ = 0°, 30° and 60° were used for calibration.

For probe calibration, the linear Stokes vectors ([*I Q U*]) exiting out of the distal end of each channel were measured with an external Thorlabs polarimeter, to determine the system matrix ***Z*** in Eq. (9). Then a 2-inch-diameter linear polariser oriented horizontally (θ = 0°) followed by a front-surface-coated mirror were placed perpendicularly to the probe end, as depicted in Fig. 3. The 635 nm laser was coupled to the H_1_ fibre, thus passing through the H_1_ micro-polariser and the external horizontal polariser, reflecting off the mirror, traversing through the external horizontal polariser again, and entering the H_2_ channel where its intensity was recorded. This is the H_1_H_2_ measurement through a 0° polariser double-pass. This was repeated for 2 additional angles of the external polariser (θ = 30°, 60°). These steps were then repeated for the V_2_ and B_1_ collection channels to generate the H_1_V_2_ and H_1_B_1_ measurements. All steps were then repeated for illumination through the V_2_ channel, and finally for the illumination through the B_1_ channel, to generate the remaining V_2_H_1_, V_2_V_1_, V_2_B_1_, B_1_H_1_, B_1_V_2_, and B_1_B_2_ measurements for the three double-pass polariser angles. The entire calibration procedure took ~30 minutes. For clinical implementations*, in vivo* probe calibration and tissue measurements will be done much more quickly (~ milliseconds-seconds) to overcome probe and patient motion; this is discussed further in Section 4. Note that all probe fibres were kept immobile during all measurements, as only the laser input and detector connections were switched at the proximal end of the probe.

For these calibration measurements, the “sample Mueller matrix” ***M***_***SAMPLE***_ was known to be that of polariser double-pass (at its three different examined angles θ = 0°, 30°, 60°), specifically:

12 $${{\boldsymbol{M}}}_{{\boldsymbol{SAMPLE}}}={{\boldsymbol{M}}}_{{\boldsymbol{POLARISER}}{\boldsymbol{AT}}{180}^{{\bf{o}}}-{\boldsymbol{\theta }}}\,\cdot {{\boldsymbol{M}}}_{{\boldsymbol{MIRROR}}}\cdot {{\boldsymbol{M}}}_{{\boldsymbol{POLARISER}}{\boldsymbol{AT}}{\boldsymbol{\theta }}}$$

Note that on the second pass through the polariser, its orientation angle becomes 180° − θ. This is due to the change in reference frame that occurs when light switches direction upon reflection at the mirror. *M*_*POLARISER AT θ*_ and *M*_*POLARISER AT 180−θ*_ were measured independently with a free-space Thorlabs polarimeter, and the mirror was assumed to be described by:

13 $${{\boldsymbol{M}}}_{{\boldsymbol{MIRROR}}}=\,[\begin{array}{ccc}1 & 0 & 0\\ 0 & 1 & 0\\ 0 & 0 & -1\end{array}].$$

We then solved for the c_1_-c_9_ coefficients via ‘best fit’ nonlinear regression^20^, by numerically minimizing the mean absolute difference between the elements of the normalized Mueller matrix as measured by the probe (using Eq. (11)), and the ‘true’ ***M*** of the polariser-mirror-polariser system (Eq. (12)). The resultant calibration “c” coefficients that scale the H_1_H_2,_ H_1_V_2,_ H_1_B_1_ V_2_H_1,_ V_2_V_1,_ V_2_B_1,_ B_1_H_1,_ B_1_V_2,_ and B_1_B_2_ measurements are: 1.18, 0.83, 0.74, 0.70, 1.21, 1.51, 1.24, 1.17, and 1.42. The standard deviation of the coefficients about the mean value (1.1 ± 0.3) represents the various probe manufacturing imperfections, as well as the partially polarised nature of the light emanating from the laser that seeds the system. Note that these non-ideal numbers represent the first probe prototype (and are adequately dealt with by the calibration procedure); subsequent probe implementation and the use of an unpolarised light source will likely make these coefficients approach unity. This may even potentially obviate the need for such extensive calibration altogether.

This completes the probe calibration. We now proceed with measurements of unknown samples in order of increasing complexity: (1) linear polariser double pass, at a θ-orientation different from the three calibration angles, (2) QWP double pass, and (3) biological tissue in reflection mode, in its native and stretched (birefringence-modulated) state.

### Data Availability Statement

The data generated and analysed during the current study are available from the corresponding author upon reasonable request.

## Results and Discussion

(1) The probe was first used to determine the Mueller matrix of a linear polariser double pass, oriented at

**Figure 4:**
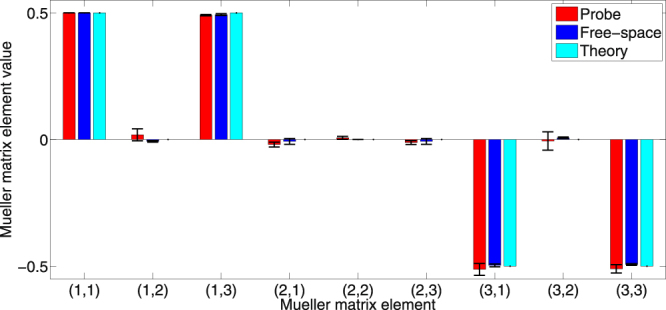
Mueller matrix elements measured by probe (red), a free-space polarimetry system (blue), and corresponding theoretical values (cyan) of a θ = 45° linear polariser double pass. Error bars indicate the standard deviation of 3 independent measurements. In between each of the 3 measurements, the sample was repositioned and the probe was recalibrated. No significant differences in matrix elements between the probe and free-space Mueller matrices were noted. See Table 1 for corresponding probe and free-space element values.

θ = 45° using the same set-up as depicted in Fig. 3. Again, the ‘true’ Mueller matrix was determined with free-space Thorlabs polarimeter measurements and ideal ***M***_***MIRROR***_ (Eq. (13)) using Eq. (12). Three measurements were made using both the fibre probe and the free-space system. The results are summarized numerically in Table 1, and graphically in Fig. 4. Agreement is seen for all nine ***M*** elements with no statistically significant differences between probe and free-space matrix elements (p ≫ 0.05 for all 2 sample, unpaired, 2 -sided t-tests performed for each element).

**Table 1:** Top row: (1) Probe *vs*. independently measured Mueller matrix of a 45° linear polariser double pass.

***(1) M*** of 45° linear polariser double pass (probe)	Independent check with free-space polarimetry
$$[\begin{array}{ccc}0.50\pm 0.00 & 0.02\pm 0.02 & 0.49\pm 0.00\\ -0.02\pm 0.01 & 0.01\pm 0.01 & -0.01\pm 0.01\\ -0.51\pm 0.02 & -0.01\pm 0.04 & -0.51\pm 0.02\end{array}]$$	$$[\begin{array}{ccc}0.50\pm 0.00 & -0.01\pm 0.00 & 0.49\pm 0.00\\ -0.01\pm 0.01 & 0.00\pm 0.00 & -0.01\pm 0.01\\ -0.50\pm 0.01 & 0.01\pm 0.00 & -0.49\pm 0.00\end{array}]$$
***(2) M*** of 45° quarter wave plate double pass (probe)	Independent check with free-space polarimetry
$$[\begin{array}{ccc}1.00\pm 0.00 & -0.07\pm 0.03 & -0.03\pm 0.08\\ 0.06\pm 0.03 & -0.91\pm 0.08 & 0.04\pm 0.17\\ -0.07\pm 0.02 & 0.19\pm 0.06 & -1.08\pm 0.04\end{array}]$$	$$[\begin{array}{ccc}1.00\pm 0.00 & 0.00\pm 0.04 & 0.01\pm 0.03\\ 0.06\pm 0.08 & -0.93\pm 0.04 & -0.10\pm 0.04\\ 0.01\pm 0.02 & 0.05\pm 0.01 & -1.02\pm 0.02\end{array}]$$

**Figure 5:**
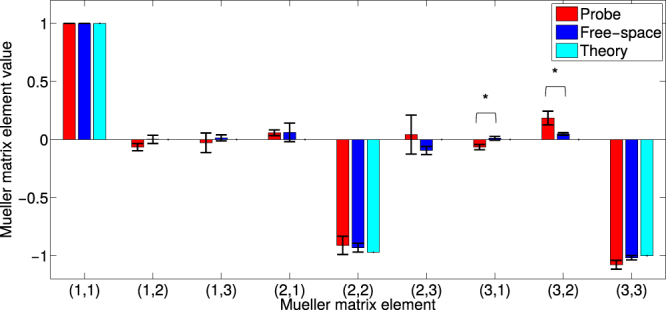
Mueller matrix elements measured by probe (red), a free-space polarimetry system (blue), and corresponding theoretical values (cyan) of a 45° quarter wave plate double pass. Error bars indicate the standard deviation of 3 independent measurements. In between each of the 3 measurements, the sample was repositioned and the probe was recalibrated. There are small but statistically significant differences in the *m*_*31*_ and *m*_*32*_ matrix elements (p = 0.009 and 0.017 respectively, calculated with an unpaired, 2-sided t-test). See Table 1 for corresponding probe and free-space element values.

See Fig. 4 for graphical representation. Bottom row: (2) Probe *vs*. independently measured Mueller matrix of QWP double pass whose fast axis was oriented at 45°. See Fig. 5 for graphical representation. The errors are the standard deviation from 3 independent measurements. In between each of the 3 measurements, the sample was repositioned and the probe was recalibrated.

Notably, the average of both free-space and probe Mueller matrix measurements of the 45° polariser double pass agree well with the theoretical matrix elements, with very small deviations. While the probe performance and accuracy are thus very encouraging, this represents the least-complex test case, in that the examined sample was not very different from those used for calibration: a linear polariser but at a new, unknown angle. We now move on to the slightly more challenging test of measuring ***M*** of a new polarising optical element altogether (while still retaining the calibration measurement geometry of Fig. 3).

(2) Next the probe was used to measure the Mueller matrix of a QWP double pass, oriented at ~45°. The results are shown in Table 1 and graphically in Fig. 5.

The theoretical Mueller matrix of a QWP double pass with fast axis at 45° and linear retardance δ = 0.27 λ is:

14 $${{\boldsymbol{M}}}_{{\boldsymbol{QWP}}{\boldsymbol{DOUBLE}}{\boldsymbol{PASS}}}=\,[\begin{array}{ccc}1 & 0 & 0\\ 0 & -0.97 & 0\\ 0 & 0 & -1\end{array}].$$

For a QWP, elements *m*_*22*_, *m*_*23*_, *m*_*32*_, and *m*_*33*_ are all sensitive to fast axis alignment θ and retardance δ. Thus, these may be affected by the difference in effective retardances, due to the probe’s high NA fibres. Additionally, sensitivity to fast axis orientation angle means any small experimental (~1°) difference in θ will influence results; both of these dependencies may be responsible for the slight inter-results variation and statistical differences between probe and free-space values of these elements.

Further, elements *m*_*12*_, *m*_*13*_, *m*_*21*_, *m*_*31*_ (first row and column) should all theoretically be zero; both sets of measurements are, but with small deviations. These may be due to small diattenuation of the QWP. The fact that slightly non-zero elements are observed in both the probe and free-space measurements supports this hypothesis. Antireflection coating on the QWP may lessen this effect in the future.

The measured *m*_*33*_ elements are slightly less than -1 for both systems. The source of this slight discrepancy is currently being investigated, and could be caused by small laser power or polarisation fluctuations during the measurement procedure, coupled with set-up errors inherent in manual fibre switching in this prototype data acquisition procedure. We are currently adapting a 4 × 4 Mueller matrix filtering technique^21^ to the 3 × 3 probe methodology to minimize these small deviations.

Looking at Figs 4 and 5, we see that the accuracy of the 45° QWP (Fig. 5) is very good, but not at the level of the 45° polariser (Fig. 4). As the QWP is more temperature and path length-sensitive than the polariser, the difference in accuracy is somewhat anticipated. Nevertheless, since no wave plates were used in the probe calibration, the probe’s performance in adequately measuring its Mueller matrix is promising and further suggests the validity of our methodology.

(3) Finally the probe was used on a more intrinsically complex biological sample of chicken breast tissue, to determine its Mueller matrix before and after it was stretched along the direction of its muscle striations. Mechanical stretching induces increased tissue anisotropy (an increase in optical birefringence), for example as shown by Alali *et al*. in *ex vivo* distended rat bladders^22^. The tissue was fixed in 4% paraformaldehyde for 24 hours, changing its polarisation properties slightly^23^, but enabling much easier tissue handling, stretching, and measurement. The ~2 mm thick fixed chicken breast slice was clamped to a home-built stretching device and positioned at the location of the mirror (Fig. 3) to approximate the calibration conditions; in real clinical scenarios, much smaller calibration and measurement working distances are envisioned. Following its Mueller matrix measurements, the tissue was stretched by ~20% along its muscle fibre direction, repositioned in the probe polarimeter setup, and re-measured. This was repeated on three additional chicken breast tissue samples.

The results are displayed in Table 2. The Mueller matrices were decomposed^24^ to determine the chicken breast’s linear retardance magnitude before and after the axial stretch (20% strain). Since reflected light undergoes a helicity flip, the measured Mueller matrix will contain effects of both the true physical retardance, as well as a geometrical retardance arising from the directional change in light propagation. This effect has been studied in non-depolarising media^25^. In the case of a wave plate of arbitrary retardance with its fast or slow axis oriented vertically (similar to this stretched tissue scenario), the physical retardance, δ_phys_, is equal to 180° − δ_meas_, where δ_meas_ is the measured retardance. (For example, the physical retardance of an ideal 30° retarder double pass should be δ_phys_ = 2 × 30° = 60°. However, δ_meas_ = 120°. Thus δ_phys_ is found by 180° − δ_meas_ = 60°. The same trend occurs for all retarders with fast or slow axis oriented vertically). As such, we report the physical tissue retardance as 180° − δ_meas_.

**Table 2:** Representative chicken breast tissue Mueller matrices pre- and post axial stretch, and mean linear retardance magnitude and depolarisation for four independently measured tissue samples.

	**Chicken breast tissue**	**Stretched chicken breast tissue**
**Representative Mueller matrix from one tissue sample**	$$[\begin{array}{ccc}1.00 & 0.36 & -0.05\\ 0.11 & 0.09 & 0.22\\ 0.11 & -0.23 & 0.54\end{array}]$$	$$[\begin{array}{ccc}1.00 & 0.38 & -0.06\\ -0.00 & -0.01 & 0.20\\ 0.02 & -0.35 & 0.22\end{array}]$$
**Mean linear retardance magnitude (degrees)**	103 ± 7	120 ± 9
**Mean depolarisation (%)**	44 ± 7	50 ± 12

The error represents the standard deviation of four independent measurements, each on different chicken breast samples. The mean linear retardance increased by ~15% (p = 0.03), while the slight mean depolarisation increase did not reach statistically significant levels (p = 0.40). P-values were calculated by an unpaired, 2-sided t-test.

As seen below, the resultant mean linear retardance due to strain increases by ~15% (from 103° to 120°) for the four chicken breast tissue samples. At a given wavelength, retardance is proportional to the product of sample thickness and its birefringence; the samples naturally become slightly thinner with stretching, and their birefringence increases as well. These two mechanisms can have opposite effects on retardance when measured in transmission geometry, and can in fact be decoupled (for example by an independent thickness measurement). For *in vivo* applications, likely in retro-reflection geometry, sampled tissue thickness cannot be directly measured. In this case, we can either utilize polarisation-sensitive Monte Carlo simulations to estimate the polarisation sampling volumes (which yields the mean pathlength and thickness estimate)^15^ or compare relative values of different linear retardance measurements (without decoupling the separate birefringence or thickness contributions)^26^. Other groups have reported birefringence increases between normal and stretched chicken breast tissue^27,28^, but the quantification of the specific dependence of birefringence on strain (~20% in this case) is unknown, likely varies for different tissue types, and makes quantitative comparisons difficult. But certainly the probe-measured trend of increasing tissue asymmetry upon stretching (as reported by birefringence and/or retardance) makes sense. Thus, the probe’s ability to detect and quantify potentially important changes in tissue polarisation properties such as those present between healthy and pre- pathological/pathological tissue (e.g., retardance contrasts between necrotic and viable breast cancer^29^, normal bladder outlet and partial bladder outlet obstruction^30^, and cervical cancer^2^) is encouraging.

Another interesting metric that naturally emerges from the Mueller matrix decomposition is tissue depolarisation (last row of Table 2). This is also seen to increase upon stretching, although more measurements are needed to properly test statistical significance. Similar findings of higher depolarisation in more birefringent tissue have been observed by Alali *et al*.^31^ where despite having the same transport albedo as porcine kidney cortex, the depolarisation of porcine myocardium muscle was ~25% higher when measured in a free-space backscattering geometry. As in the retardance/birefringence discussion above, the slight tissue thickness decrease upon stretching is again a confounder that would likely contribute to decreased depolarisation; it appears that for these samples, the increased birefringence may have played a larger role in the resultant depolarisation trend.

**Figure 6:**
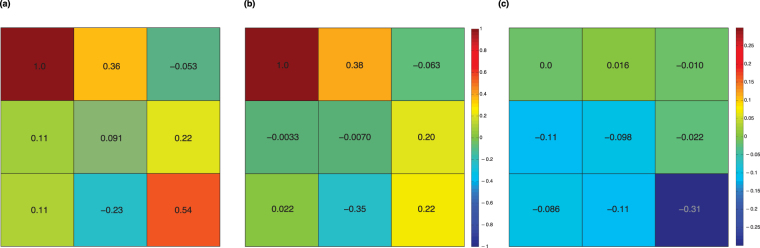
Heat maps of (**a**) normal and (**b**) stretched 3 × 3 Mueller matrices for one representative chicken breast tissue (see Table 2); (**c**) displays their difference. Upon stretching, most elements decrease except *m*_12_. The *m*_33_ element exhibits the largest decrease (~60%), as previously observed in birefringent polyacrylamide phantoms upon stretching^32^.

To further explore these results graphically, we display one tissue sample’s representative Mueller matrices (as in Table 2) via colour maps, and show their difference. Figure 6 shows that upon stretching, most elements slightly decrease in value except a slight increase in *m*_*12*_ (*m*_*11*_ naturally stays fixed because of normalization). The four lower left elements decrease more than the three upper righthand elements, but the *m*_*33*_ element decreases in magnitude the most (~60%). Such large relative decrease in *m*_*33*_ magnitude has also been observed in controlled birefringent polyacrylamide phantoms upon stretching^32^.

As evidenced by the presented results, the flexible probe design enables accurate Mueller matrix measurements of an ideal linear polariser and of an ideal retarder (quarter-wave plate). In Mueller matrix polarimetry of birefringent chicken breast, the probe detects an expected increase in strain-induced optical anisotropy between the normal and stretched tissue states. Encouraged by our demonstrated ability to detect and quantify changes in *ex vivo* tissue, we are currently progressing to *in vivo* studies to distinguish between healthy and pathologic (or pre-pathologic) tissue regions that likely exhibit birefringence and depolarisation contrasts^3,29^.

### Engineering Design Considerations and Biological Context

Having successfully demonstrated proof-of-concept of the *flexible* polarimetric methodology, there are several areas of performance improvements to consider for its next prototype. Further, a practical and robust calibration procedure suitable for *in situ* implementation must be developed. The improvements include (1) using an opaque component holder material: in the presented probe, MED610 was used which is a readily available, translucent, and biocompatible 3D printing material. However, its translucency may have permitted some cross talk between the probe channels, so an optically opaque material will be used in the next prototype. (2) Improving resolution via tip sculpting optics: the distal tip of the probe could be enhanced using fibre bevelling or attaching ball lenses^33^ that can steer the illumination and collection apertures toward each other, as such, optimizing the interplay between resolution, working distance, overlap sampling volume, and light collection efficiency. Making use of the central channel will also be helpful in this regard. (3) Incorporating optical circulators: these will enable illumination and collection of light through the same channel, which increases overlapping sampling volumes while also reducing the needed number of fibre channels, total probe diameter, variance in illumination intensity, and acquisition time. (4) The next probe prototype will have QWPs, enabling full 4 × 4 Mueller matrix determination. Obtaining such a complete polarisation signature of the sample is important since the additional elliptical/circular polarisation information can reveal even more significant biophysics, for example as related to the detection of chiral glucose molecules^19^. Glucose monitoring is impossible with a 3 × 3 approach but may be possible via 4 × 4 Mueller polarimetry^15,34^. Further, the complete Mueller matrix determination will also improve the accuracy of the linear polarisation metrics over the partial 3 × 3 formalism^35^, as well as furnish an unambiguous birefringence orientation value (e.g., a 0° QWP and a 90° QWP have the same 3 × 3 Mueller matrix). The probe would require 2 additional fibres (if no optical circulators were used) to illuminate and collect circularly polarised light, each equipped with distal polariser and QWP; with circulators, the minimum number of fibres required would be reduced to 4. Finally, (5) a depolarised light source will be used in place of the current partially polarised diode laser, to minimize the resultant inter-channel intensity modulation effects. In practice for *in situ* calibration, partial reflectors or a small, switchable mirror (liquid crystal or mechanical) will be mounted at the distal end of the probe. First the probe will be guided to the region of interest via conventional white-light endoscopy. Then for the active opto-electronic control embodiment, the crystal will switch electronically to reflective mode (or mechanically move into the beam path), intensity measurements will be made to generate calibration coefficients, then the crystal will revert to transparency mode (or mechanically move out of the beam path), and the 9 (or 16 for the full 4 × 4 prototype) tissue intensity measurements will be made. *In situ* calibration must be performed rapidly prior to tissue measurements, to avoid probe and tissue motion artefacts. Programmable optoelectronics and the proposed use of circulators will reduce the requisite acquisition time to milliseconds-seconds range. The engineering details and measurement protocols of this implementation are currently being investigated. High-heat autoclaves will be avoided in favour of chemical disinfection between uses, to preserve the integrity of the distal optics, and possibly opto-electronics^36^.

We envision the probe to be used as a flexible, polarisation-resolved add-on to human endoscopes, with access through the latter’s working/biopsy channels. For example, one application we are currently pursuing is polarisation cystoscopy in urology, with the intent to aid in the treatment guidance of partial bladder outlet obstruction (pBOO). Upon obstruction in rats, Alali *et al*. found that there were significant regional changes in optical anisotropy (birefringence) of the bladder wall, particularly the ventral region close to the urethra^30^. The probe could be used to pinpoint such local regions of high linear retardance changes and optimize surgical procedures (bladder augmentation) in pBOO on an individual patient basis, a vast improvement when compared to the current practice that treats the upper (dome) region of the bladder exclusively^37^.

Another unmet urological need calls for improved detection of early stage bladder cancer, as the current standards of white light cystoscopy, narrow-band imaging, and photodynamic diagnosis (“blue light” cystoscopy) have a high false positive rate^38^. Changes in the bladder extracellular matrix have been successfully detected with polarised light in *ex vivo* rat studies of pBOO^30^; it is thus likely that *in vivo* flexible cystoscopic probe polarimetry may help detect pre-cancerous remodelling^39^ of the extracellular matrix in humans. Other applications for flexible polarisation endoscopy include probing the uterine cervix^40^ and colon for detection of precancerous sites^41^, and liver for improved liver fibrosis diagnosis^42^.

## Conclusion

We have demonstrated a flexible, 6-fibre probe with distal micro-polarisers capable of accurate 3 × 3 linear Mueller matrix measurement of polarisation components (polarisers, wave plates) and of birefringent biological tissues. We described the probe manufacturing, its calibration and sample data acquisition procedures, and initial performance characterization in controlled polarisation-optics samples and in *ex vivo* biological tissues. Several areas of improving the proposed flexible polarimetric methodology have been outlined. Overall, the demonstrated ability to accurately determine the 3 × 3 Mueller matrix of biological tissues via a flexible fibre optic probe bodes well for eventual full polarimetric examinations of difficult-to-reach clinical body sites and tissues.
